# Distance learning in antimicrobial stewardship: innovation in medical education

**DOI:** 10.1186/s12909-019-1623-x

**Published:** 2019-06-07

**Authors:** Michel Laks, Carla Morales Guerra, João Luiz Miraglia, Eduardo Alexandrino Medeiros

**Affiliations:** Infectious Diseases Division, Department of Internal Medicine, Escola Paulista de Medicina, Universidade Federal de São Paulo, Napoleão de Barros, 690, second floor, São Paulo, Brazil

**Keywords:** Antimicrobial stewardship, Distance education, Medicine education

## Abstract

**Background:**

This study aimed to analyse the impact that web-based distance learning has on knowledge gain in medical students, as well as student perceptions of the methodology.

**Methods:**

This was an educational intervention study conducted at a tertiary teaching hospital in the city of São Paulo, Brazil. From 2008 to 2014, we offered a free web-based distance learning course, covering antimicrobial use and microbial resistance, to fifth-year medical students. The course encompassed 100 h of activities, with five theoretical modules, exercises and simulations, within a virtual learning environment. The students were tutored in their online activities, and some classes were conducted in real time for live discussions. In addition, students underwent face-to-face assessments of their knowledge of the topic before and after the course. Statistical analysis was performed and the means of the overall scores were obtained, as were the respective 95% confidence intervals (CIs). The means were compared by two-tailed paired *t*-tests and by the paired Wilcoxon test.

**Results:**

Of 814 eligible medical students, 606 (74.45%) completed the entire course during the study period. The mean score for knowledge of the topic was significantly higher on the final assessment than on the initial assessment (*p* <  0.001). We found that dedication (in hours) was directly proportional to the level of participation, as reflected in the mean final score (*p* = 0.009) and in the proportion of students who passed (*p* = 0.028). All of the participants considered their knowledge adequate or insufficient before the course, stating that it is quite important or important to address the topic during medical education. Although dedication levels were low, 70.5% stated that they had learned “quite a lot” or “more than expected” about the topic and would dedicate more time to it if they could.

**Conclusions:**

The use of a virtual learning environment can promote teaching and learning in the infectious diseases field, specifically for antimicrobial stewardship, increasing knowledge significantly, and should be considered for inclusion in the final stages of medical education.

## Background

Over the years, the field of teaching has involved various approaches and different methodologies, evolving from a rigid structure with passive methods to a permissive scenario with active methods that are more constructive [1]. The difficulty of incorporating new knowledge into an already saturated pedagogical plan in a medicine course has prompted a search for new methodologies, as well as the use of innovations in information and communication technologies [2]. Among the tools used, the Modular Object-Oriented Dynamic Learning Environment (Moodle) platform, a collaborative virtual learning environment (VLE), is increasingly more widely used by international health promotion agencies and educational institutions, especially in programmes that encompass the topic of antimicrobial therapy. The importance of the topic stems from the fact that antimicrobial agents affect not only the individual but also the environment, altering the sensitivity profiles of bacteria and causing the emergence of antimicrobial resistance. In the twenty-first century, antimicrobial resistance has had a significant impact on the quality of health care and on patient safety. That, combined with a rapidly dwindling antimicrobial armamentarium, has resulted in a critical threat to public health worldwide, especially in developing countries such as Brazil.

Antimicrobial stewardship refers to coordinated interventions designed to quantify and improve the appropriate use of antimicrobial agents, promoting the selection of the optimal antimicrobial drug regimen, dose, duration of therapy and route of administration. Antimicrobial stewards seek to achieve optimal clinical outcomes related to antimicrobial use; minimise toxicity and adverse events; reduce the costs of health care for infections; and limit the selection of antimicrobial resistant strains.

Antimicrobial stewardship programmes optimise antimicrobial use to achieve the best clinical outcomes minimising adverse events and limiting selective pressures that drive the emergence of resistance, as well as reducing the costs attributable to suboptimal use of antimicrobial agents. Therefore, antimicrobial stewardship is a fiduciary responsibility for all healthcare facilities across the continuum of care, making it essential that initiatives for its instruction be implemented.

To date, there have been few reports of the experiences of medical schools in assessing knowledge gains related to the use of methodologies such as VLEs in topics within the field of infectious diseases. At most medical schools in Brazil, the pedagogical plan does not include addressing the problem of microbial resistance or the techniques of antimicrobial stewardship. The use of a VLE can fill that gap and could provide a benefit to the health care system when medical graduates begin their professional practice. The objective of this study was to analyse the incorporation of a distance learning environment (DLE) for antimicrobial use and the prevention of antimicrobial resistance into the curriculum of one of the largest medical schools in Latin America and to evaluate its impact on the acquisition of knowledge about the subject, as well as to collect student perceptions of the methodology.

## Methods

We conducted this study under the auspices of the Hospital Epidemiology Committee in the Infectious Diseases Division of the Department of Medicine of the *Escola Paulista de Medicina/Universidade Federal de São Paulo* (EPM/Unifesp, Federal University of São Paulo Paulista School of Medicine). The EPM/Unifesp operates the Hospital São Paulo, a tertiary care facility with 740 beds, as a venue for the practical activities of medical students, in the city of São Paulo, which is the capital of the state of São Paulo, the most populous state in Brazil. [3] Approximately 730 medical students are currently enrolled in the EPM/Unifesp, which has an average annual enrolment of 121 students, selected through one of the most competitive college entrance examinations in the country. Unifesp is one of the main universities in Brazil, as reflected in the national and major international university rankings [4–6].

This was an educational intervention study in which we developed and implemented a Moodle-based distance learning course on the use of antimicrobial agents and the prevention of antimicrobial resistance. We evaluated the course over seven consecutive years (2008–2014). In the first year of the study (2008), fifth- and sixth-year medical students (those in their next-to-last and last years of medical school, respectively) took the course, whereas we offered it only to fifth-year students thereafter. The study was conducted in four phases: development of the theoretical content and virtual interfaces; application of the course; data collection and analysis of the performance and impressions of the participating students; and statistical analysis of the results obtained.

The course was offered to students via e-mail and through the use of posters placed around the university campus. It was specified that it was an extra-curricular (not-for-credit) course. The students took the course on a voluntary basis, and all participating students gave written informed consent. The questionnaires were made available online, and the responses were kept confidential. Participant privacy was maintained during and after the study period, the data being used exclusively for the purposes of the study. The study was approved by the Research Ethics Committee of the Hospital São Paulo (Protocol no. 429,036).

We used content previously produced for an antimicrobial stewardship/antimicrobial resistance prevention DLE that employed a Moodle-based VLE and was offered to health care professionals [7, 8]. Within that context, the theoretical content was made available via a user-friendly interface, as were the links to the collaborative activities. This process of development and application of the course was carried out by tutors throughout the year, approximately 54 h of development activities and 30 h of activities being employed in its application. Those activities included monitoring student logs in real time, the tutors encouraging the students to face the problems, resolve questions and make the correct clinical decision. In each year of the study, three face-to-face meetings were held in order to discuss the course with the participants. The course was programmed for 100 h of activities—including theoretical foundations (the only part of the course that was publicly accessible), on-line exercises/simulations and in-person activities and was composed of five modules. Module 1 presented the antimicrobial agents and concepts related to their use in general, as well as to each antimicrobial class, each antimicrobial agent being described in terms of their pharmacological characteristics, mechanisms of action, clinical indications, dosages and adverse reactions. Module 2 presented the main mechanisms of antimicrobial resistance developed by bacteria. Module 3 helped the students correctly interpret microbiology data, which are essential in clinical practice. Module 4 presented the main community-based and health care-associated infections, as well as their treatment. Finally, Module 5 listed the main measures to prevent and control infection with antimicrobial-resistant microorganisms. In addition to the theoretical content, each module included interactive simulations of clinical cases, in which each student received feedback on the alternative selected; a forum for discussion and clarification of questions; and a questionnaire on the content studied.

There were two forms of evaluation. First, a written questionnaire, devised by the tutors and based on the course content, was applied to evaluate participant knowledge before the beginning of the course (“initial face-to-face assessment”), and, after the students had been monitored throughout the course under a tutelage system, the written questionnaire was again applied (“final face-to-face assessment”). The results of the initial and final face-to-face assessments were compared by statistical methods in order to assess the knowledge gained by the students. The second form of evaluation was the application of questionnaires related to each VLE module. In both forms of evaluation, the theoretical concepts of all five modules were addressed, as were aspects related to their clinical application; a passing score was defined as ≥7 (out of 10). All of the data collected were stored in databases.

### Statistical analysis

We calculated the proportion of students who completed the initial and final assessments, that of those who completed only the initial assessment and that of those who completed neither. Additional analyses included only the students who completed both assessments.

The normality of the distribution of the initial and final assessment scores, collectively, was assessed using the Mann-Whitney test for Skewness and Kurtosis. All analyses of the score dataset were carried out in two phases: including and excluding the sixth-year students, who took part only in 2008. The means of the overall scores were obtained, as were the respective 95% confidence intervals (CIs). The means were compared by two-tailed paired *t*-tests.

The proportional distributions of the responses to questions related to prior antimicrobial knowledge (initial assessment questions) and to evaluation of the course (final assessment questions) were determined for the sample as a whole. Bar graphs were created to illustrate those distributions. Statistical analysis was performed with the Stata statistical software package, version 10.1 (StataCorp LP; College Station, TX, USA).

## Results

The total numbers and proportions of students who enrolled in the course, of those who enrolled and attended the initial assessment but did not engage in the VLE activities, of those who enrolled but did not attend the initial assessment or engage in the VLE activities and of those who completed the course are shown in Table 1, by year. Between 2008 and 2014, a total of 606 students completed the course: 72 were sixth-year students (2008 only); and 534 were fifth-year students (2008–2014). Beginning in 2013, there was a downward trend in the number of students enrolled in the course, as well as in the proportion of students who completed it.

**Table 1:** Enrolment and participation in the EPM/Unifesp antimicrobial stewardship course, 2008–2014

Year*	Enrolled	Participation		
		Full (completed)	Initial assessment only	None
	*N*	*n* (%)	*n* (%)	*n* (%)
2008				
Fifth-year students	108	90 (83.3)	18 (16.7)	0
Sixth-year students	72	72 (100.0)	0	0
2009	123	118 (95.6)	5 (4.0)	0
2010	117	116 (99.1)	1 (0.9)	0
2011	117	93 (79.5)	21 (18.0)	3 (2.5)
2012	129	73 (56.6)	31 (24.0)	25 (19.4)
2013	85	19 (22.4)	66 (77.6)	0
2014	63	25 (39.7)	37 (58.7)	1 (1.6)

*Except where otherwise indicated, all enrollees were fifth-year medical students

The overall (initial plus final assessment) mean scores, with and without the scores of the sixth-year students, are shown in Table 2. The means for the datasets including and excluding the scores of the sixth-year students were virtually identical. The paired *t*-test showed that the mean score was significantly higher in the final assessment than in the initial assessment (*p* <  0.001 for both tests). A qualitative analysis of these evaluations allows us to affirm that the students had greater difficulty in answering the questions related to Modules 1, 3 and 4. However, because the tests are not standardized, it is not possible to attribute that greater difficulty exclusively to the difficulty of the specific theme of a given Module. Notably, Modules 1 and 4 were those that attracted the most attention from the participants, perhaps due to the high clinical applicability of the concepts presented therein.

**Table 2:** Mean scores in the initial and final assessments, 2008–2014

Sample	N	Initial assessment	Final assessment	p*
		Mean (95% CI)	Mean (95% CI)	
All students	606	5.78 (5.70–5.86)	8.45 (8.36–8.53)	< 0.001
All fifth-year students	534	5.75 (5.66–5.83)	8.46 (8.37–8.54)	< 0.001

^*^Two-tailed paired *t*-test

The mean scores of the sixth-year students were significantly higher (with no overlap in the 95% CIs) than were those of the fifth-year students who took the course in the same year (2008), not only in the initial assessment (6.02 vs. 5.54; *p* < 0.001) but also in the final assessment (8.40 vs. 7.77; *p* = 0.002).

The proportion of students who had a passing grade was significantly higher in the final assessment than in the initial assessment, whether or not the grades of the sixth-year students were included (*p* < 0.001 for both). The difference between the two was considerable: 10.9% (with or without the grades of the sixth-year students) in the initial assessment, compared with 86.6 and 87.6% (with and without the grades of the sixth-year students, respectively) in the final assessment. Analysing the years collectively, we found that dropping out was a major cause of failure to pass the course, accounting for 25.55% of the failures.

On average, the students with a higher self-reported level of participation scored higher on the final assessment (8.75; 95% CI: 8.59–8.91) than did those reporting a lower level of participation (8.48; 95% CI: 8.34–8.61), and the difference was statistically significant (*t*-test, *p* = 0.009). The proportion of students with a passing grade in the final assessment was also significantly higher among those reporting a higher level of participation (94.8%; 95% CI: 89.1–98.1 vs. 87.1%; 95% CI: 81.8–91.4, *p* = 0.028). Although the 95% CIs overlapped slightly in both cases, the hypothesis tests showed that the differences were statistically significant.

**Fig. 1:**
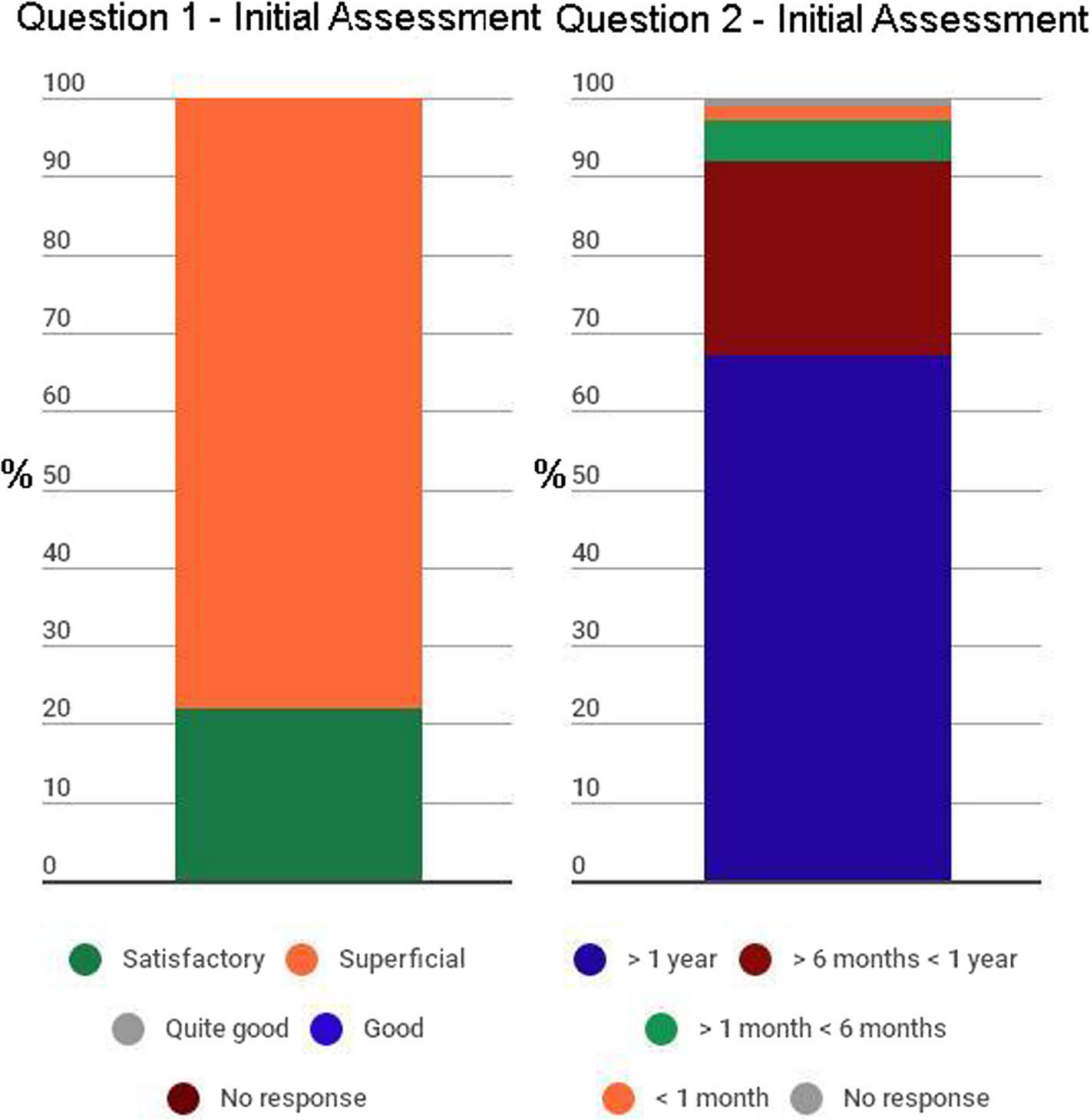
Proportional distribution of responses to questions 1 and 2 on the initial assessment, 2009–2014. Legend: Question 1: How would you classify your knowledge of antimicrobial agents?; Question 2: When was your last formal update (course, lecture or bibliographic search) on the topic of antimicrobial agents?

Figure 1 shows the proportional distribution of responses on the initial self-report assessment of student knowledge about antimicrobial agents. None of the students initially categorised their knowledge of antimicrobial agents as very good or even good, and approximately two thirds had received their last formal update on the topic more than one year prior.

**Fig. 2:**
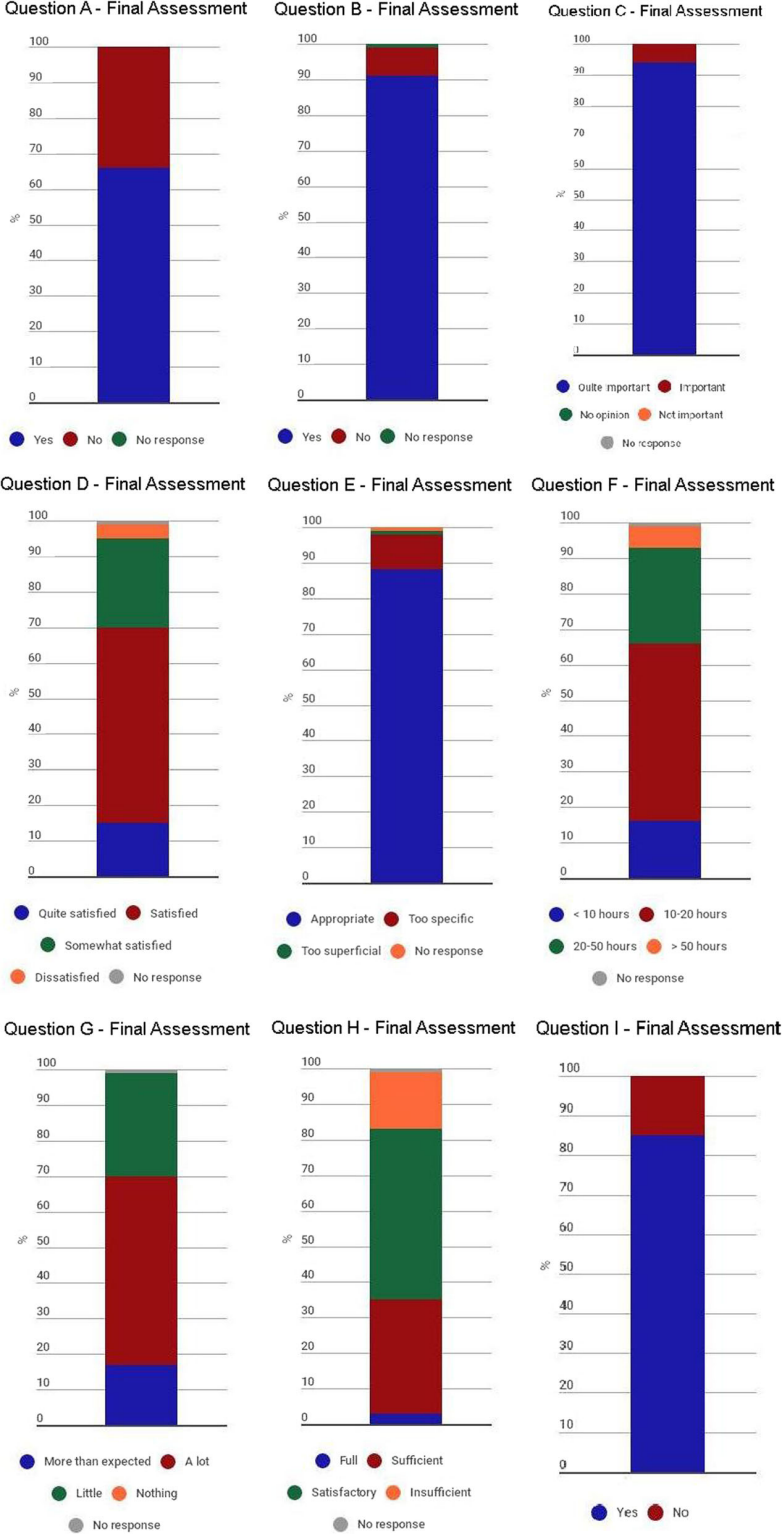
Proportional distribution of responses to questions on final course, 2009, 2011, 2012, 2013 and 2014. Legend: Question A: Was this you first online course?; Question B: Would you take another distance learning course coordinated by the Unifesp Infectious Diseases Division?; Question C: How important do you think it is to include the topic of the rational use of antimicrobial agents in the curriculum of a medical school?; Question D: Overall, how satisfied are you with the course?; Question E: What is your opinion about the content of the course?; Question F: On average, how many hours did you dedicate to the course, including online and offline studies?; Question G: How much do you think you have learned in this course?; Question H: How would you categorise the level of your participation in the course?; Question I: If you had had more time, do you think you could have dedicated yourself more to this course?

Figure 2 shows the proportional distribution of responses on the final self-report assessment, in which the students evaluated the course, quantified the time they had dedicated to it, and qualified their participation in it. Approximately two thirds of the students reported that the course represented their first interaction with a VLE, and 556 (91.8%) stated that they would want to take another course organised by the Infectious Diseases Division of the EPM/Unifesp Department of Medicine. Regarding the topic of the course, 567 (93.6%) of the students considered the “rational use of antimicrobial agents” important enough to be included in the medical curriculum. In their evaluation of the course content, 534 (88.1%) of the students found it to be appropriate, whereas 63 (10.4%) considered it to be too specific for a medical school course. In addition, 421 (69.5%) classified themselves as satisfied or very satisfied with the course, whereas 183 (30.2%) expressed some degree of dissatisfaction. In their evaluation of the knowledge gain resulting from the course, 421 (69.5%) of the students reported that they had learned a lot or more than they had expected about the topic. The time dedicated to the course was also evaluated: Approximately half of the participants estimated that, between online and face-to-face activities, they had dedicated 10–20 h to the course, whereas 5.80% reported that they had dedicated more than 50 h to it. From the analysis of the student logs, we inferred that the level of interest in the activities proposed in the VLE was similar for each type of activity (e.g., real time discussions and online simulations). Of the 606 students who completed the course, 215 (35.4%) qualified the level of their participation as full or sufficient. In addition, 516 students (85.1%) stated that they would have dedicated more time to the course if they had been able to.

## Discussion

Fifth-year medical students were offered a course, based on a DLE strategy, on the use of antimicrobial agents and the prevention of microbial resistance. The objective of this study was to determine whether there was a quantitative knowledge gain and thus whether this teaching modality can be considered valid. To encourage students to engage with the topic, the course was in compliance with the Unifesp institutional guidelines.

Beginning in 2013, there was a downward trend in the number of students enrolled in the course, as well as in the proportion of students who completed the course. This trend might be due to the formula and presentation becoming played out. To remedy that situation, the content was reformulated and the course was validated as an elective course, students who completed the course earning credits. Those measures also have been sufficient to increase the number of students enrolling in the course since 2015.

The fact that the mean final scores were significantly higher than the mean initial scores supports the main hypothesis of the study. Our finding that dropping out was a major cause of failure to pass the course corroborates data from the 2014 Brazilian DLE Census [9]. We believe that the causes of dropout in the present study were similar to those previously reported in the literature [10], such as a lack of time to study or participate in the course due to other curricular or extra-curricular activities.

Our results, like those of other studies of distance learning initiatives in the field of infectious diseases [11], are indicative of validity and the favourable cost-benefit ratio of the method. More than being just an option, a DLE can represent the only applicable methodology or the one that has the most favourable cost-benefit ratio, especially in resource-poor locales or in those that are far from educational centres (for example, a university with distant campuses, where there is a need to deliver unique content), making it an interesting alternative [12–19].

As previously mentioned, a portion of the students expressed some degree of dissatisfaction with the course, and some adjustments therefore needed to be made. The reported reasons are in agreement with the findings of other studies of such initiatives [9, 20]. One adjustment that could be made is the optimisation of the relationship between the volume of content and the time allotted, as suggested by Premkumar et al. [20] However, as stated by Groenwold et al. [21], it is also necessary to allow students the freedom to manage their own curricular and extra-curricular activities in a competent manner. That equation can be tenuous and needs to be individualised, the course tutelage program playing a role in improving student achievement.

This study has some limitations. First, the questionnaires employed in the initial and final assessments were not standardised across the years evaluated. The study design could also be considered a limitation, because there was no control group or randomisation. The timing of the course within the medical education curriculum is also another possible point of distortion in the results: offering the course to sixth-year, rather than fifth-year, medical students could have yielded results that were worse (given that sixth-year students are more intently focussed on their curricular activities and on studying for the residency examination) or better (because sixth-year students ostensibly possess more knowledge than do fifth-year students and can use the course as a form of study for the residency examination). Another limitation of the study is the fact that, in the face-to-face assessments, we used questions that were quite similar to those employed in the evaluations of the VLE. Finally, it should be noted that, in the analyses of the questions assessing previous knowledge, course participation and perceptions of the course, statistical calculations were not always carried out.

An interesting topic for discussion is whether distance learning can replace the traditional methodology. The positive results we obtained for knowledge gain, together with the varied perceptions of the students, allow us to conclude that the DLE is not a replacement for the traditional strategy but can be adopted as a complement, and that instructors should shift the focus toward one or the other depending on the level of development, cognitive gain and preferences of the students, as well as on how the students deal with new technologies. Studies comparing distance learning and traditional methodologies have produced conflicting results, some favouring distance learning [21, 22] and others indicating that the two are equivalent [23–25], as in the systematic review recently conducted by Ahmadi et al. [25] However, there are important subjective aspects that are not evaluated in that type of comparison. To elucidate this issue, studies that evaluate it in a more objective manner are needed.

Another important question is whether and to what degree a distance learning methodology can have a positive influence on antimicrobial prescription practices when the student becomes a physician and begins to practice medicine. Unfortunately, the basic guidelines for the proper use of antimicrobial agents, measures for the prevention of nosocomial infection and antimicrobial resistance are not part of the pedagogical plan at most medical schools. In general, medical school curricula are heavily loaded with content and concepts, leaving no space to introduce fundamental concepts related to the proper prescription of antimicrobial agents, which is important regardless of which specialty the students choose in the future. Extra-curricular activities within a DLE could help medical schools bridge that gap.

The U.S. Centers for Disease Control and Prevention (CDC) recommends some core elements of antimicrobial stewardship, one of which is “education” [26]. In their joint guidelines on the implementation of an antibiotic stewardship program [27], the Infectious Diseases Society of America and the Society for Healthcare Epidemiology of America advocated for teaching hospitals to integrate the fundamental principles of antibiotic stewardship into their curricula. Although the CDC recommendation is related to providing instruction for health professionals, we believe that this core element should be extended to medical school curricula.

Our findings support the assertion that the strategy of offering a VLE-based distance learning course is a valid means of increasing student knowledge about antimicrobial stewardship, as suggested by Fakih et al. [28] Although there are no data in the literature with a high grade of recommendation for the use of distance learning as a strategy for teaching topics within the infectious diseases field to medical students, it is believed that this is attributable to the scarcity of studies on the subject and to the different methodologies employed—a situation described in 2003 by Christante et al. [29] and that seems to persist—and it is therefore difficult to make a uniform assessment. Therefore, the present study represents an important attempt to provide a more scientific basis for the use of this strategy.

## Conclusions

This study suggests that distance learning can replace the traditional methodology in a complementary way, because there is significant improvement on learning process. The students, overall, approved the method, but should have dedicated more time to the course if they had been able to. New approaches and programmatic contents are necessary to avoid dropout, allowing students the freedom to manage their own curricular and extra-curricular activities in a competent manner. We recommend that initiatives like this be used in order to instruct medical students in how to apply antibiotic stewardship to their activities in teaching hospitals.

## Data Availability

The datasets used and analysed during the current study are available from the corresponding author on reasonable request.

## Abbreviations

CDC: Centers for Disease Control and Prevention
CI: confidence intervals
DLE: distance learning environment
EPM/Unifesp: Escola Paulista de Medicina/Universidade Federal de São Paulo
Moodle: Modular Object-Oriented Dynamic Learning Environment
QS: Quacquarelli Symonds
Unifesp: Universidade Federal de São Paulo
VLE: virtual learning environment
